# Effects of BMP2 and VEGF165 on the osteogenic differentiation of rat bone marrow-derived mesenchymal stem cells

**DOI:** 10.3892/etm.2013.1464

**Published:** 2013-12-27

**Authors:** ZHAOWEI LIN, JIANG-SHENG WANG, LIJUN LIN, JINGWEN ZHANG, YUNLONG LIU, MING SHUAI, QI LI

**Affiliations:** 1 Department of Orthopaedics, Zhujiang Hospital, Southern Medical University, Guangzhou, Guangdong 510282, P.R. China; 2 Department of Orthopaedics, Lund University Hospital, S-22185 Lund, Sweden; 3 Department of Orthopaedic Rehabilitation, Guangdong Provincial Work Injury Rehabilitation Center, Guangzhou, Guangdong 510440, P.R. China

**Keywords:** bone marrow-derived mesenchymal stem cells, bone morphogenetic protein 2, vascular endothelial growth factor, bone regeneration, co-transfection

## Abstract

Bone marrow-derived mesenchymal stem cells (MSCs) are dominant seed cell sources for bone regeneration. Bone morphogenetic proteins (BMPs) initiate cartilage and bone formation in a sequential cascade. Vascular endothelial growth factor (VEGF) is an essential coordinator of extracellular matrix remodeling, angiogenesis and bone formation. In the present study, the effects of the vascular endothelial growth factor 165 (VEGF165) and bone morphogenetic protein 2 (BMP2) genes on bone regeneration were investigated by the lentivirus-mediated cotransfection of the two genes into rat bone marrow-derived MSCs. The successful co-expression of the two genes in the MSCs was confirmed using quantitative polymerase chain reaction (qPCR) and western blot analysis. The results of alizarin red and alkaline phosphatase (ALP) staining at 14 days subsequent to transfection showed that the area of staining in cells transfected with BMP2 alone was higher than that in cells transfected with BMP2 and VEGF165 or untransfected control cells, while the BMP2 + VEGF165 group showed significantly more staining than the untransfected control. This indicated that BMP2 alone exhibited a stronger effect in bone regeneration than BMP2 in combination with VEGF165. Similarly, in inducing culture medium, the ALP activity of the BMP2 + VEGF165 group was notably suppressed compared with that of the BMP2 group. The overexpression of VEGF165 inhibited BMP2-induced MSC differentiation and osteogenesis *in vitro*. Whether or not local VEGF gene therapy is likely to affect bone regeneration *in vivo* requires further investigation.

## Introduction

The development of gene therapy and transgenic technology has provided an effective means with which to treat a variety of diseases (1). With regard to non-union fractures and bone defects, bone regeneration is a major challenge. *Ex vivo* gene therapy has been demonstrated to strengthen bone regeneration by guiding osteogenic differentiation (2).

Bone morphogenetic proteins (BMPs), a group of secreted proteins that belong to the transforming growth factor-β (TGF-β) superfamily, initiate cartilage and bone formation in a sequential cascade (3). Among the BMP family members, BMP2 has the highest osteoinductive activity *in vivo* (4). Although osteogenic factor BMP2 is important during the induction of bone formation, bone regeneration is a highly complicated process that involves a number of growth factors. Vascular endothelial growth factor (VEGF), the most well-characterized angiogenic factor, is crucial in bone healing and skeletal development (5). BMP-induced VEGF production in osteoblast-like cells is important in the coupling of bone formation and angiogenesis (6).

Since BMP2 and VEGF are involved in bone formation, it has been proposed that the combined delivery of BMP2 and VEGF may have a more effective impact on bone regeneration than the delivery of a single gene alone. Furthermore, the specific roles of BMP2 and VEGF in the process of bone formation require further investigation. In order to elucidate the efficacy of the combination of the two growth factors, a constructive gene therapy model was created using Sprague Dawley (SD) rat bone marrow-derived mesenchymal stem cells (MSCs) that were lentivirally cotransfected with hBMP2 and hVEGF165. The expression of bone regeneration was then investigated using this model.

## Materials and methods

### Isolation and expansion of rat MSCs

The bone marrow was obtained from purchased rats. After the rats were sacrificed, the femur and tibia were separated, both ends of each bone were snipped and bone marrow was washed with 10 ml Dulbecco’s modified Eagle’s medium (DMEM) culture medium twice. The bone marrow cells were cultured in tissue culture dishes (BD Falcon™; BD Biosciences, Franklin Lakes, NJ, USA) in DMEM supplemented with 10% (v/v) fetal bovine serum (FBS) and 1% (w/v) penicillin/streptomycin at 37°C in 5% CO_2_. Since the MSCs were able to adhere to the surface of culture dishes (whereas hemopoietic cells were not), the adherent cells were isolated from the bone marrow through adherence-separation culturing, with the medium changed twice a week. Upon reaching 80% confluency, the cells were detached using 0.25% (w/v) trypsin/1 mM EDTA solution (1:1, v/v), replated at a density of 1×10^4^ cells/cm^2^ in tissue culture dishes and cultured as first-passage cells (P1) until confluency (5–7 days). MSCs of passage 3 (P3) were used for transfection.

### Lentiviral vector construction and production

The cDNAs for BMP2, VEGF165 and enhanced green fluorescent protein (EGFP), obtained from Cyagen Biosciences, were amplified using the polymerase chain reaction (PCR) with the primers listed in Table I. Following this, the BMP2-EGFP and BMP2-VEGF165-EGFP cDNAs were subcloned into the pLV-EX2d-EF1A expression lentivector (Invitrogen Life Technologies, Carlsbad, CA, USA), respectively, and 293FT producer cells were cotransfected with pLV/helper packaging plasmid mix (Invitrogen Life Technologies) and expression lentivector (containing BMP2-EGFP or BMP2-VEGF165-EGFP) plasmid using Lipofectamine™ 2000 (Invitrogen Life Technologies). Thus, the lentivirus containing BMP2-EGFP cDNA (Lv-BMP2-EGFP-Neo) and the lentivirus containing BMP2-VEGF165-EGFP cDNA (Lv-BMP2-VEGF165-EGFP-Neo) were obtained.

**Figure 1:**
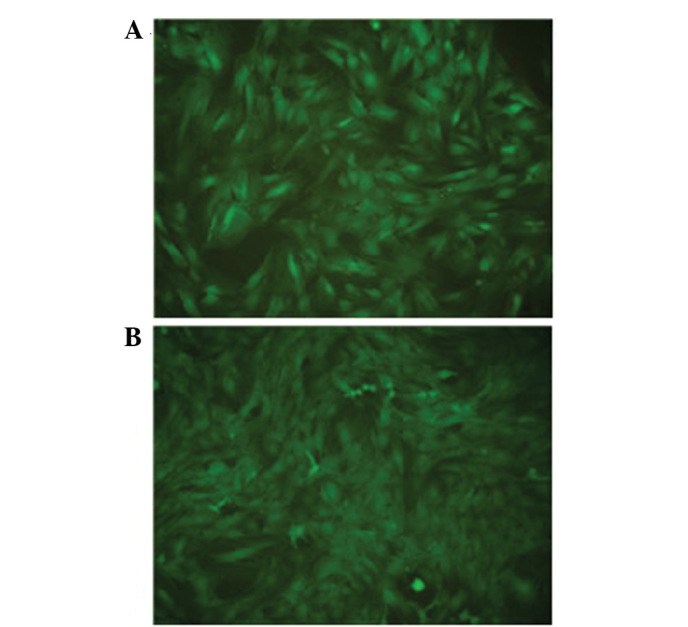
Rat mesenchymal stem cells (MSCs) in culture (passage 5) were transfected with (A) Lv-BMP2-EGFP-Neo or (B) Lv-BMP2-VEGF165-EGFP-Neo at a multiplicity of infection (MOI) of 20. A transfection efficiency of <90% was obtained, as determined by the fraction of fluorescent cells using fluorescent microscopy 2 days subsequent to the transfection. BMP, bone morphogenetic protein 2; EGFP, enhanced green fluorescent protein; VEGF165, vascular endothelial growth factor 165.

Cultured rat MSCs of passage 5 were transfected with Lv-BMP2-EGFP-Neo and Lv-BMP2-VEGF165-EGFP-Neo at a multiplicity of infection (MOI) of 5, 10 and 20, respectively. The efficiency of the lentiviral gene transfer in the MSCs was quantitatively determined according to the fraction of fluorescent cells using fluorescence microscopy, in accordance with the manufacturer’s instructions (Invitrogen Life Technologies) at 2 days subsequent to transfection. The fraction of cells that glowed green and reflected the lentiviral gene transfer efficiency was dose-dependent in the range of 5–20 MOI. The highest transfection efficiency, of <90%, was obtained at an MOI of 20. There were three groups in this study: MSCs infected with Lv-BMP2-EGFP-Neo (BMP2 group) at 20 MOI (Fig. 1A), MSCs infected with Lv-BMP2-VEGF165-EGFP-Neo (BMP2 + VEGF165 group) at 20 MOI (Fig. 1B) and untransfected control.

### Western blot analysis of BMP2 and VEGF

SD rat bone marrow-derived MSCs were transfected with Lv-BMP2-EGFP-Neo and Lv-BMP2-VEGF165-EGFP-Neo virus and incubated for 48 h. The transfected and untransfected SD-MSCs were lysed with radioimmunoprecipitation assay (RIPA) buffer with phenylmethylsulfonyl fluoride (PMSF) on ice for 5 min, and the total protein of the sample was assessed. A total of 100 μg protein was suspended in 5X sodium dodecyl sulfate (SDS) buffer and subjected to SDS-polyacrylamide gel electrophoresis (PAGE) using 8% Tris-HCl gel. The separated proteins were subsequently transferred to a polyvinylidene difluoride (PVDF) transfer membrane. The membranes were blocked for 1 h at room temperature in Tris-buffered saline with casein. Antibodies against BMP2 or VEGF monoclonal immunoglobulin G (IgG; dilution, 1:250; Santa Cruz Biotechnology, Inc., Santa Cruz, CA, USA) were incubated on the membrane overnight at 4°C and detected using secondary horseradish peroxidase-conjugated antibodies (dilution, 1:2,500; Santa Cruz Biotechnology, Inc.) mixed at room temperature for 1 h. The PVDF transfer membrane was then washed with Tris-buffered saline and developed using an enhanced chemiluminescence detection system with exposure to FluorChem™ HD2 (Cell Biosciences, Inc., Santa Clara, CA, USA).

### Quantitative reverse transcription-polymerase chain reaction (qPCR) analysis

Gene-specific primers for VEGF165, BMP2 and glyceraldehyde-3-phosphate dehydrogenase (GAPDH) were designed as the primer set (Table II) to detect the relative mRNA expression. The MSCs were collected respectively from the BMP2 and BMP2 + VEGF165 groups described previously at 5 days subsequent to transfection, with untransfected cells as a control. Total RNA was extracted using the TRIzol method (Ambion, Austin, TX, USA) from each cell sample and the cDNA was synthesized from total RNA. qPCR was performed using SYBR-Green^®^ Realtime PCR Master mix (Toyobo, Osaka, Japan). To correct for differences in the RNA quality and quantity among the samples, the data were normalized to the data for GAPDH.

### Alkaline phosphatase (ALP) and alizarin red staining

ALP (BCIP/NBT staining buffer) and alizarin red staining were performed in each group (control, BMP2 and BMP2 + VEGF165 groups) at 14 days subsequent to osteogenic differentiation using osteogenic differentiation medium (R&D Systems, Minneapolis, MN, USA). The samples were observed under a microscope (Olympus IX5; Olympus Corp., Tokyo, Japan).

### Analysis of ALP activity

The cells of each group were analyzed 3, 7 and 14 days subsequent to cell seeding for osteogenic differentiation. The ALP activities in the cells were assessed using an ALP detection kit (Nanjing Jiancheng Biotechnology Ltd., Nanjing, China). The absorbance of the *p*-nitrophenol was quantified using an enzyme-linked immunosorbent assay (ELISA) plate reader at a wavelength of 495 nm, by correlating the fluorescence with *p*-nitrophenol content using standards containing 0.1 mg/ml *p*-nitrophenol.

### Statistical analysis

The results are expressed as the mean ± standard deviation (SD). The statistical analysis was conducted using analysis of variance (ANOVA). P<0.05 was considered to indicate a statistically significant difference.

## Results

### qPCR

**Figure 2:**
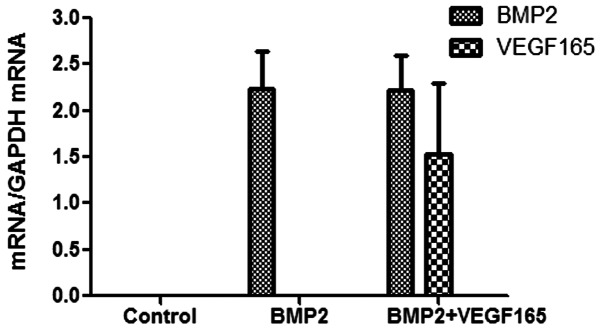
Relative mRNA expression of bone morphogenetic protein 2 (BMP2) and vascular endothelial growth factor 165 (VEGF165) in mesenchymal stem cells (MSCs) for each group (control, BMP2 and BMP2 + VEGF165) at 5 days subsequent to transfection. The results are expressed as a ratio to the mRNA levels of the reference gene, glyceraldehyde-3-phosphate dehydrogenase (GAPDH). Data are expressed as the mean ± standard deviation. (n=3 for each group).

The qPCR results are shown in Fig. 2. The mRNA expression levels of the BMP2 and VEGF165 genes in the control group were undetectable. VEGF165 was immeasurable in the BMP2 group; however, VEGF165 expression was detected in the BMP2 + VEGF165 group. With regard to BMP2 expression, BMP2 expression was detected in the BMP2 and the BMP2 + VEGF165 group. Moreover, no significant difference in the level of BMP2 expression was shown between the BMP2 + VEGF165 and BMP2 groups (P>0.05).

### Western blot analysis of BMP2 and VEGF165 secreted from the transfected MSCs

**Figure 3:**
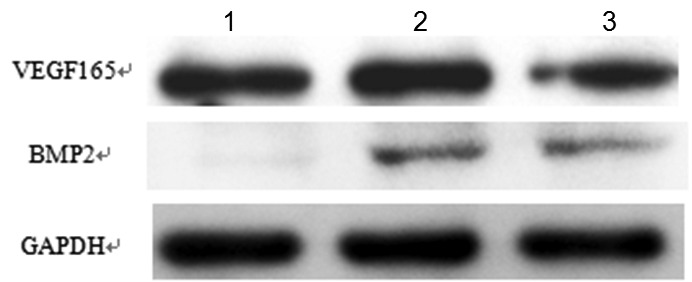
Western blot analysis of the secretion of bone morphogenetic protein 2 (BMP2) and vascular endothelial growth factor 165 (VEGF165) in transfected Sprague Dawley mesenchymal stem cells (SD-MSCs). Lane 1, SD-MSC; lane 2, SD MSC/BMP2-VEGF165; lane 3, SD MSC/BMP2.

Western blot analysis indicated that the SD-MSCs which were transfected with Lv-BMP2-VEGF165 or Lv-BMP2 secreted large quantities of BMP2; however, the SD-MSCs which were not transfected secreted low quantities, as shown in Fig. 3. By contrast, in all of the groups, the SD-MSCs secreted large quantities of VEGF, irrespective of whether they were transfected with Lv-BMP2-VEGF165 or Lv-BMP2, or were not transfected.

### ALP and alizarin red staining

**Figure 4:**
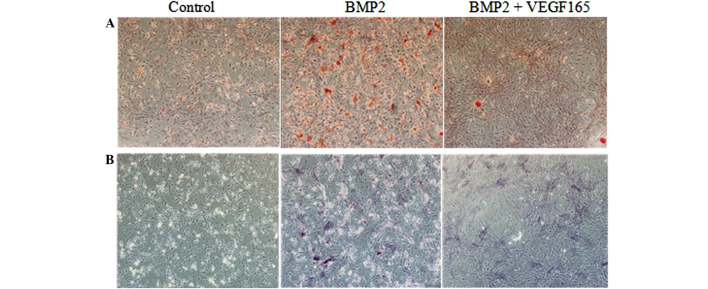
(A) Alizarin red and (B) alkaline phosphatase (ALP) staining at 14 days subsequent to transfection of mesenchymal stem cells (MSCs) in the control, bone morphogenetic protein 2 (BMP2) and BMP2 + vascular endothelial growth factor 165 (BMP2 + VEGF165) groups.

The results of alizarin red and ALP staining are shown in Fig. 4. The data indicated that the area exhibiting positive staining in the BMP2 group was significantly the largest among the three groups at 14 days subsequent to transfection. Furthermore, the area that exhibited positive staining in the BMP2 + VEGF165 group was significantly greater than that in the control group, although it was not as large as that in the BMP2 group. The alizarin red and ALP staining revealed negative results in the control group.

### ALP activity assay

**Figure 5:**
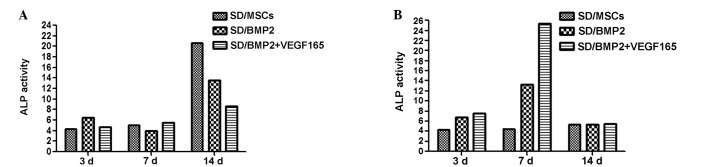
Analysis of alkaline phosphatase (ALP) activity for each group [control, bone morphogenetic protein 2 (BMP2) and BMP2 + vascular endothelial growth factor 165 (BMP2 + VEGF165)] at 3, 7 and 14 days subsequent to culturing with (A) inducing medium and (B) normal medium. The results are shown as the mean ± standard deviation (n=5 for each group). SD, Sprague Dawley; MSCs, mesenchymal stem cells.

The ALP activity results at 3, 7 and 14 days subsequent to culturing the cells with increasing of inducing medium and normal medium are shown in Fig. 5A and B, respectively. With inducing medium, the ALP activity was enhanced at 14 days in all groups compared with that at 3 and 7 days (P<0.01). At 14 days, the ALP activity of the BMP2 + VEGF165 group was notably suppressed compared with that of the BMP2 group (P<0.01). However, there were no differences between the groups at 3 and 7 days (Fig. 5A).

In the normal medium culture, the ALP activity was significantly enhanced in the BMP2 + VEGF165 group following 7 days incubation (P<0.01); however, this increase in activity was not apparent at 3 and 14 days. In a comparison between the BMP2 and BMP2 + VEGF165 groups, the ALP activity was significantly enhanced in the BMP2 group at 7 days (P<0.01; Fig. 5B).

## Discussion

Bone tissue engineering is a potential pathway for bone regeneration, and bone marrow-derived MSCs are dominant seed cell sources for bone engineering. MSCs possess the capability to differentiate into bone, cartilage, muscle and fat when supplied with nutrition and different types of growth factors (8). Establishing a stabilized surrounding environment, which enables tissue formation, delays the ageing process of seeding cells and promotes the activities of cell proliferation and differentiation, is crucial, whether the process occurs *in vivo* or *in vitro*. The development of gene therapy and transgenic technology has provided a viable means with which to tackle the previously mentioned problems (9).

BMPs regulate bone and cartilage differentiation by the isolation, cloning and expression of genes (8). Lieberman *et al* (10) demonstrated that BMP2-producing cells, via adenoviral gene transfer, produced sufficient protein to heal segmental bone defects. The present study also indicates the significance of using MSCs transduced with BMP2 for the repair of segmental defects. Compared with the use of MSCs alone, bone regeneration is accelerated by BMP2-expressing MSCs.

BMP2 has been shown to be unable to increase the rate of bone healing, due to inadequate vascularization in certain critical-sized bone defects (11). Previous studies have shown that, following treatment with BMP2, 25% of non-union fractures required a secondary bone graft procedure, due to the lack of adequately vascularized tissue (12,13). Moreover, Furumatsu *et al* (14) demonstrated that vascularization is a critical problem in tissue engineering.

VEGF has been characterized as a heparin-binding angiogenic factor with specific mitogenic actions on endothelial cells (15). VEGF, which induces endothelial cell proliferation, angiogenesis and capillary permeability, is produced in a regulated manner by osteoblasts (16). VEGF-A has four isomers, which are composed of 121, 165, 189 and 206 amino acids, respectively. Among these isomers, VEGF165 has the most potent activity. A previous study only used the isomer of VEGF that resulted the weakest induced activity (17). In the present study, the VEGF165 isomer was used, which has been shown to lead to the most efficacious induction in activity. The process of bone metabolism includes bone formation and resorption, which are regulated by osteoblasts and osteoclasts, respectively. It has been demonstrated that VEGF is an essential coordinator of extracellular matrix remodeling, angiogenesis and bone formation in the growth plate (18). However, the role of VEGF production in osteoblasts has recently been widely discussed. Schönmeyr *et al* (19) revealed that VEGF was a potent inhibitor of BMP2 expression in MSCs, and that supplementation with or overexpression of VEGF inhibited BMP2 mRNA expression, protein production and MSC differentiation. In the present study the most positive alizarin red and ALP staining results were in the BMP2 group, which showed that the effect of BMP2 on bone regeneration was stronger than that of BMP2 and VEGF165 combined. The result suggested that the overexpression of VEGF inhibited the action of BMP2 in osteogenesis *in vitro*. Similarly, in the inducing culture medium, the ALP activity of the BMP2 + VEGF165 group was notably suppressed compared with that of the BMP2 group. However, the inhibition occurred at 14 days, which was inconsistent with the results of a previous study, in which the inhibition occurred at 21 days (20). This inconsistency may be due to a number of reasons, including cell line differences. We propose that the primary reason was that VEGF required interactions with various factors involved in bone formation to induce a greater effect. Song *et al* (21) indicated that the regulation of Id1 expression by VEGF and BMP2 may be critical to cell and gene-based approaches for bone regeneration. However, the specific role of VEGF in bone healing has yet to be elucidated. The ALP activity was higher at 14 days in inducing medium compared with that in normal medium, which indicated that there may be certain compositions that are able to promote the induction of rat bone marrow MSC differentiation.

In conclusion, the present study, which constructed BMP2 and VEGF-modified bone for tissue engineering through lentiviral transfection, revealed important implications for novel therapeutic strategies to enhance bone regeneration. BMP2 was shown to be important in bone regeneration in the *in vitro* study. The overexpression of VEGF inhibited BMP2-induced MSC differentiation and osteogenesis *in vitro*. Whether or not local VEGF gene therapy is likely to affect bone regeneration *in vivo* has yet to be elucidated. A further study is ongoing.

## Figures and Tables

**Table I:** Primers of VEGF165 and BMP2 genes from Cyagen Biosciences for the amplification of VEGF165 and BMP2 by PCR.

Gene	Primer
attB1-Kozak-BMP2-F	GGGGACAAGTTTGTACAAAAAAGCAGGCTGCCACCATGGTGGCCGGGACCCGC
BMP2-T2A-R-1	AAGACTTCCCCTGCCCTCTCCGGAGCCGCGACACCCACAACCCTCC
T2A-VEGF165-F	CGGGGACGTGGAGGAAAATCCCGGCCCCATGAACTTTCTGCTGTCTTGGGTG
VEGF165-P2A-R-1	ACAGAGAGAAGTTCGTGGCGCCGCTGCCCCGCCTCGGCTTGTCACAT
P2A-EGFP-F	GCAAGCAGGAGATGTTGAAGAAAACCCCGGGCCTATGGTGAGCAAGGGCGAGGA
T2A-EGFP-F	CGTGGAGGAAAATCCCGGCCCCATGGTGAGCAAGGGCGAGG
attB2-EGFP-R	GGGGACCACTTTGTACAAGAAAGCTGGGTTTACTTGTACAGCTCGTCCATG

VEGF165, vascular endothelial growth factor 165; BMP2, bone morphogenetic protein 2; PCR, polymerase chain reaction; EGFP, enhanced green fluorescent protein.

**Table II:** Gene-specific primers for VEGF165, BMP2 and GAPDH were designed as the primer set for qPCR analysis to detect the relative mRNA expression.

Gene	Primers
BMP2-F	CGTCAAGCCAAACACAAACAGC
BMP2-R	GAGCCACAATCCAGTCATTCCAC
VEGF165-F	GCCTTGCTGCTCTACCTCCAC
VEGF165-R	GCACACAGGATGGCTTGAAGATG
GAPDH Rat-F	CCTTCCGTGTTCCTACCC
GAPDH Rat-R	CAACCTGGTCCTCAGTGTAG

VEGF165, vascular endothelial growth factor 165; BMP2, bone morphogenetic protein 2; GAPDH, glyceraldehyde-3-phosphate dehydrogenase; qPCR, reverse transcription-polymerase chain reaction.
